# A step-by-step guide for implementing alcohol screening & brief intervention in primary care: pilot & evaluation in three U.S. settings

**DOI:** 10.1186/1940-0640-10-S2-O37

**Published:** 2015-09-24

**Authors:** Elizabeth Dang, Daniel Hungerford, Nancy Cheal

**Affiliations:** 1 Fetal Alcohol Syndrome Prevention Team, Centers for Disease Control and Prevention, Atlanta, USA

## Background

Risky alcohol use can cause a range of negative consequences, including motor vehicle crashes, intimate partner violence, and medical conditions such as hypertension, gastritis, liver disease, and various cancers [1]. Alcohol use during pregnancy can result in miscarriage, stillbirth, and fetal alcohol spectrum disorders (FASDs) [2,3]. Over thirty years of research has shown that alcohol screening and brief intervention (SBI) is effective at reducing risky drinking. Yet, it has not been integrated widely into clinical settings.

In 2014, CDC released *Planning and Implementing Screening and Brief Intervention for Risky Alcohol Use: A Step-by-Step Guide for Primary Care Practices*[4]. The guide provides a practical process that primary care settings can use to implement alcohol SBI. Prior to publication, a draft version of the guide was tested with three CDC-funded FASD Regional Training Centers (RTCs) to implement alcohol SBI as routine care for all adults in 10 primary care clinics in Alaska, Nevada, and Tennessee.

## Materials and methods

To assist the FASD RTCs in their implementation efforts, CDC provided them with a draft of the implementation guide. CDC hired Westat to evaluate the quality and effectiveness of the guide, using qualitative research methods, including site visit interviews with program and clinic staff.

## Results

Data were obtained from 56 RTC and clinic respondents. Results showed that the guide was most useful for initial planning, engaging key clinic stakeholders, developing training and dissemination materials, and for additional resources and references. Multiple challenges with the draft guide were identified, including the need for protocols for brief interventions and information on billing and reimbursement codes.

## Conclusions

Evaluation findings were used to revise the guide prior to its release. This presentation will highlight the evaluation, findings, modifications, and an overview of the guide as currently available from CDC.
